# Activation of the PERK-ATF4 pathway promotes chemo-resistance in colon cancer cells

**DOI:** 10.1038/s41598-019-39547-x

**Published:** 2019-03-01

**Authors:** Zhong Shi, Xiaofu Yu, Meiqin Yuan, Wangxia Lv, Tingting Feng, Rui Bai, Haijun Zhong

**Affiliations:** 1 Department of Medical Oncology, Zhejiang Cancer Hospital, Hangzhou, 310022 China; 2 Department of Radiotherapy, Zhejiang Cancer Hospital, Hangzhou, 310022 China; 3 Department of Medical Oncology, Hangzhou Cancer Hospital, Hangzhou, 310000 China

## Abstract

Colon cancer is a major health problem worldwide. While chemotherapy remains a main approach for treating late-stage colon cancer patients, most, if not all, of them will develop drug resistance and die of uncontrollable disease progression eventually. Therefore, identification of mechanism of drug resistance and development of overcoming strategy hold great significance in management of colon cancer. In this study, we discovered that activation of the PERK branch of the unfolded protein response (UPR) pathways is required for colon cancer cells to survive treatment of 5-Fluorouracil (5-FU), one of the first-line chemotherapeutics for late-stage colon cancer patients. Genetic and pharmacological inhibition of PERK or its downstream factors greatly sensitize colon cancer cells to 5-FU. Most importantly, *in vivo* use of PERK inhibitor synergizes with 5-FU in suppressing the growth of colon cancer cells in mouse models. In summary, our findings established a promising way to overcome resistance to chemotherapy in colon cancer.

## Introduction

Colorectal cancer (CRC) is the third most common cancer in the US, with over 146,000 new cases and almost 57,000 deaths each year, making it the second leading cause of death from cancer among adults^1^. Surgical resection is potentially curative for patients with local, early-stage CRC; however, surgery is not applicable for CRC patients with extensively metastatic disease and treatment options for them are very limited^2^. Currently, chemotherapy remains the mainstay for treating unresectable late-stage CRC, and fluorouracil-based regimens are most frequently used chemotherapy regimens^3^. Albeit effective early on, almost all patients will develop resistance to fluorouracil-based treatment and succumb to cancer progression^4^. Apparently, there is unmet need to resolve the adaptive resistance of CRC cells to chemotherapy.

One widely studied mechanism by which cancer cells resist therapy is through activation of a stress-adaptation program termed the unfolded protein response (UPR)^5–7^. The UPR – which is conserved across metazoa – is induced by nutrient deprivation, hypoxia, oxidative stress, viral infection and accumulation of misfolded proteins within the endoplasmic reticulum (ER)^8–10^. UPR signaling is initiated by three distinct receptors localized to the ER membrane – protein kinase RNA-like endoplasmic reticulum kinase (PERK), endoplasmic reticulum-to-nucleus signaling1 (ERN1/IRE1α), and ATF6^11–14^. While these receptors converge on multiple shared downstream signaling molecules, including BIP, CHOP and GADD34, they also have unique signaling effects: activated IRE1α induces splicing of XBP1 mRNA, resulting in the translation of a frame-shifted stable form of the protein that functions as a transcription factor (XBP1(S)); activated PERK phosphorylates eIF2α, inducing an integrated stress response associated with global translational repression and selective translation of repair proteins (e.g., ATF4). Upon activation, the ATF6 protein will be translocated to the Golgi apparatuses, and cleaved by S1P and S2P to generate a mature form of transcription factor. Activation of UPR has been shown to promote cell survival of breast, lung, and liver cancer cells, and involved in drug resistance^15–17^. However, the role of UPR in drug resistance of CRC to chemotherapy is not known.

In this study, we aimed to investigate if activation of the UPR pathways contributes to chemo-resistance of human CRC cells. By analyzing all three branches of the UPR pathway, we found that activity of the PERK-ATF4 pathway is up-regulated in CRC cells that show heightened resistance to 5-fluorouracil (5-FU). Genetic or pharmacological inhibition of the PERK-ATF4 pathway can effectively sensitize CRC cells to 5-FU treatment. Taking together, we discovered a cellular stress pathway that can confer drug resistance, and identified a potential approach to overcome chemo-resistance in human colon cancer.

## Materials and Methods

### Ethics Statement

This study was performed in strict accordance with the recommendations in the Guide for the Care and Use of Laboratory Animals of the Zhejiang Chinese Medical University. The protocol was approved by the Animal Care and Use Committee of the Zhejiang Chinese Medical University. All surgery was performed under isoflurane anesthesia, and every effort was made to minimize suffering.

### Cell lines and reagents

SW1116, LoVo, Colo320DM, SW480, SW620 and CT26 cell were from ATCC and cultured in RPMI1640 + 10% FBS. 5-FU was from Sigma. The PERK inhibitor was described previously and purchased from EMD Millipore. The IRE1α inhibitor was purchased from MCE. Lentiviral short hairpin RNA (shRNA) constructs targeting PERK, ATF4, IRE1, GCN2 and PKR were generated as described previously^18^. Lentiviral integration was selected with 2 µg/ml puromycin for 5 days.

### Cell survival analysis

Cells were plated in 100 μl of culture medium per well in 96-well plates, at a density of 2000 cells/well. 24 hrs after seeding, compounds were added at 8 different doses with three replicates per dose per cell line. The same volume of DMSO was added in three replicates per line as a control. Cell viability was measured after 72 hrs with the CellTiter-Glo Assay (Promega).

### ATF6 reporter assay

p5xATF6-GL3 and hRluc constructs were obtained from Addgene (Plasmid #11976^19^ and #24348). One day after co-transfection of 0.5 μg p5xATF6-GL3 and 0.05 μg hRluc plasmids, ATF6 activity of cells was measured by a dual luciferase assay (Promega).

### Western blot

Cultured cells were lysed on ice with cold RIPA buffer plus complete protease inhibitor cocktail (Roche Applied Science). Cell lysates were clarified by centrifugation at 12000 g for 10 min, and protein concentration was determined by the BCA Reagent. Lysates were separated on NuPAGE 4–12% Bis-Tris gel electrophoresis, proteins were then transferred to nitrocellulose membrane and immunoblotted with the following antibodies: ATF4 (Cell Signaling, 11815, 1:1000), PERK (Cell Signaling, 9956, 1:2000), GAPDH (Cell Signaling, 3683, 1:2000), p-eIF2α (Cell Signaling, 3597, 1:500), total eIF2α (Cell Signaling, 9722, 1:2000), IRE-1 (Cell Signaling, 3294, 1:1000), and spliced XBP-1 (Biolegend, 619501, 1:500). All immunoblots were visualized by enhanced chemiluminescene. Raw data of all western blots were included in Supplementary Figs S4 and S5.

### Flow cytometry analysis

Flow cytometry analysis was performed according to the manufacturer’s protocol (BD Biosciences), with at least 10000 live events captured per analysis. Cells after treatment as indicated were treated with RNase and stained with propidium iodide (5 μg/ml) (BD Bioscience). Percentage of sub-G0/G1 cells was measured to indicate apoptotic cell death.

### *In vivo* tumor growth

2 × 10^6^ SW620 cells were injected subcutaneously into 6–8-wk-old female NOD/SCID mice. After tumor reaching 60–80 mm^3^, animals were treated with PBS by IP injection, 5-FU (20 mg/kg) by IP injection, PERK inhibitor (10 mg/kg) by oral administration, or a combination of 5-FU and PERK inhibitor three times per week for 3 weeks^20^. Tumor volume over time and tumor weight at sacrifice were measured and presented as the average ± standard error of mean for 5 tumors per treatment group. All animals were randomized by weight.

### Statistical analysis

All data are presented as mean ± standard error of mean unless otherwise specified. Student t test (two-tailed) was used to compare two groups of data, and two way ANOVA was used to analyze drug treatment. *p* < 0.05 was considered significant.

## Results

### Evaluating the activity of the unfolded protein response pathways in human colon cancer cell lines

**Figure 1:**
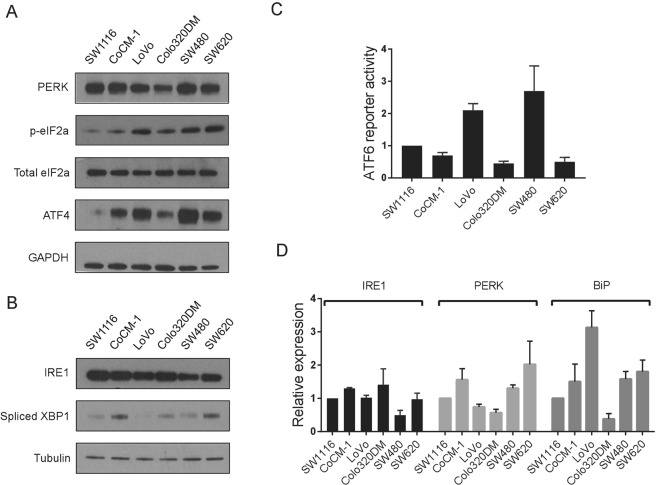
Activation of the unfolded protein response pathways in colon cancer cells. (**A**) Western blot showing the expression of the PERK pathway genes in six colon cancer cells. GAPDH was used as loading control. (**B**) Western blot showing the expression of the IRE1 pathway genes in cells in (**A**). Alpha Tubulin was used as loading control. (**C**) Luciferase reporter assay showing the transcriptional activity of ATF6 in the cells in (**A**). Activity of ATF6 was normalized to Renilla luciferase. (**D**) Realtime RT-PCR showing the relative expression level of IRE1, PERK and BiP mRNA in six colon cancer cells. GAPDH was used as loading control. Data are represented as mean ± SEM or the mean alone.

We first examined the activation of three branches of UPR pathways in six colon cancer cell lines. To assess the activity of PERK pathway, we gauged the expression level of two downstream factors of the PERK kinase, phospho-eIF2α and ATF4 (Fig. 1A,D), as well as a direct target of ATF4, GADD34 (Fig. S1A). To assess the activity of the IRE1 pathway, we measured the expression of spliced XBP1, a downstream factor of IRE1 upon its activation (Fig. 1B,D). For the ATF6 pathway, we applied a reporter-based assay to measure the transcriptional activity of ATF6, since currently there are no reliable antibodies to detect the cleavage of endogenous ATF6 protein (Figs 1C and S1A). BiP is a critical chaperon protein involved in ER stress. We found that BiP expression was also differently expressed among CRC cells (Fig. 1D). In addition to its basal level, we also measured the response of each arm of the UPR to Thapsigargin, a classical ER stressor. Similarly, the UPR pathways of CRC cells were induced differently by Thapsigargin (Fig. S1B,C). Based on the above results, we found that the UPR pathways are differentially activated in CRC cells.

### Activation of the PERK-ATF4 pathway is required for resistance of CRC cells to 5-Fluorouracil treatment

**Figure 2:**
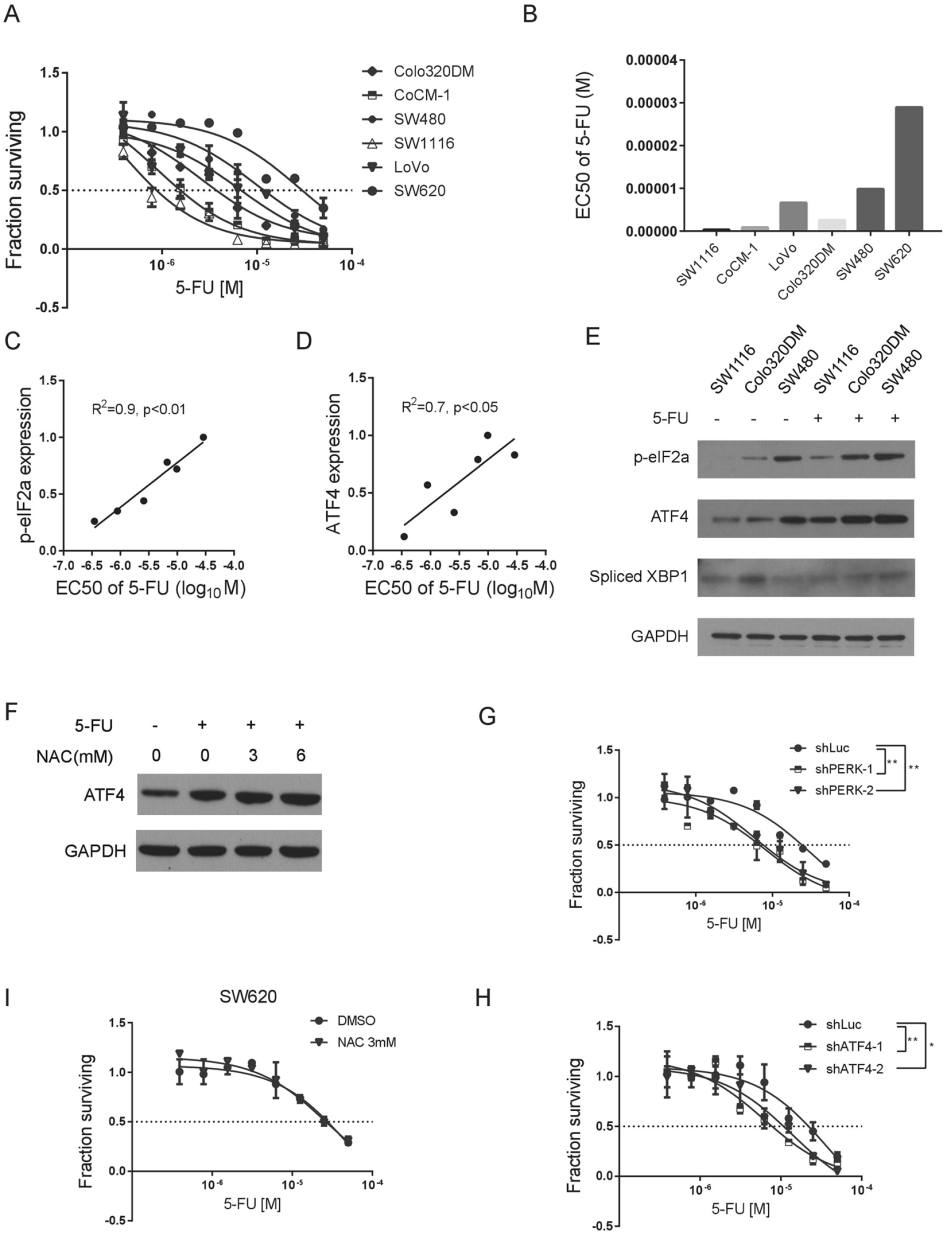
The PERK pathway correlates and is required for colon cancer cells to survive 5-Fluorouracil treatment. (**A**) Dose response curves showing responses of six colon cancer cells to treatment of 5-Fluorouracil (5-FU) treatment. (**B**) EC50 of 5-FU treatment in the six colon cancer cells. (**C**) and (**D**) Correlation analysis of the expression of phosphorylated eIF2α and ATF4 and EC50 of 5-FU in colon cancer cells. **(E)** Western blot showing the effects of 5-FU treatment on PERK-ATF4 pathway and IRE1-sXBP1 in SW1116, Colo320DM and SW480 cells. GAPDH was used as loading control. (**F**) Western blot of ATF4 showing the effects of NAC on 5-FU treated SW620 cells. (**G**) Dose response curves showing responses of SW620 cells transduced with a hairpin targeting Luciferase (shLuc) or two hairpins targeting PERK (shPERK-1, shPERK-2) to treatment of 5-FU. (**H**) Dose response curves showing responses of SW620 cells transduced with a hairpin targeting Luciferase (shLuc) or two hairpins targeting ATF4 (shATF4-1, shATF4-2) to treatment of 5-FU. (**I**) Dose response curves showing effects of the NAC (3 mM) in sensitizing SW620 cells to 5-FU treatment. * means *P* < 0.05, ** means *P* < 0.01. Data are represented as mean ± SEM or the mean alone.

To evaluate if the activity of UPR pathways is associated with the sensitivity or resistance of CRC cells to chemotherapeutic regimens, we tested the response of CRC cell lines to treatment of 5-FU. With a 3 day-treatment, the EC50 of 5-FU on CRC cells was ranged from ~200 nM to 20 µM (Fig. 2A,B). We next examined if the activation level of the UPR pathways correlates with the EC50 of 5-FU in these cells. We found that the level of phospho-eIF2α and ATF4 positively correlates the EC50, while the level of sXBP-1 (spliced XBP-1), activity of ATF6 or the expression of BiP does not show significant correlation (Figs 2C,D and S2A–C). In addition, the PERK-ATF4 pathway is induced upon 5-FU treatment (Fig. 2E), and the induction of ATF4 is not due to alteration of oxidative stress, since treatment of NAC, a reducing agent, does not reverse the increase of ATF4 upon 5-FU treatment (Fig. 2F) while IRE1-sXBP1 and ATF6 were not significantly changed (Figs 2E and S2D). These results suggested that the PERK-ATF4 branch of the UPR pathways may regulate the sensitivity of CRC cells to 5-FU treatment.

To test this hypothesis, we knocked down PERK in SW620 cells and conducted 5-FU treatment (Fig. S2E). Decrease of PERK expression significantly reduces the level of p-eIF2α, confirming the role of PERK in regulating eIF2α in CRC cells. As a control, loss of other two eIF2α kinases, GCN2 and PKR, does not change the level of p-eIF2α (Fig. S2F). We found that reduction of PERK expression can significantly sensitize SW620 cells to 5-FU treatment (Fig. 2G). Consistently, reduction of ATF4, a downstream factor of PERK, also increases sensitivity of SW620 cells to 5-FU treatment (Fig. 2H and S2G). To confirm the specific role of the PERK pathway in mediating CRC cells’ resistance to 5-FU treatment, we went on to knock down IRE1 and ATF6, respectively. We found that reduction of the IRE1 and ATF6 pathways does not alter the sensitivities of CRC cells to 5-FU treatment (Fig. S2H–K). In addition, treatment of NAC does not change the sensitivity of SW620 cells to 5-FU treatment (Fig. 2I). Collectively, activation of the PERK-ATF4 pathway is functionally required for CRC cells to resist chemotherapy.

### Chemical inhibition of PERK sensitizes CRC cells to 5-Fluorouracil treatment

**Figure 3:**
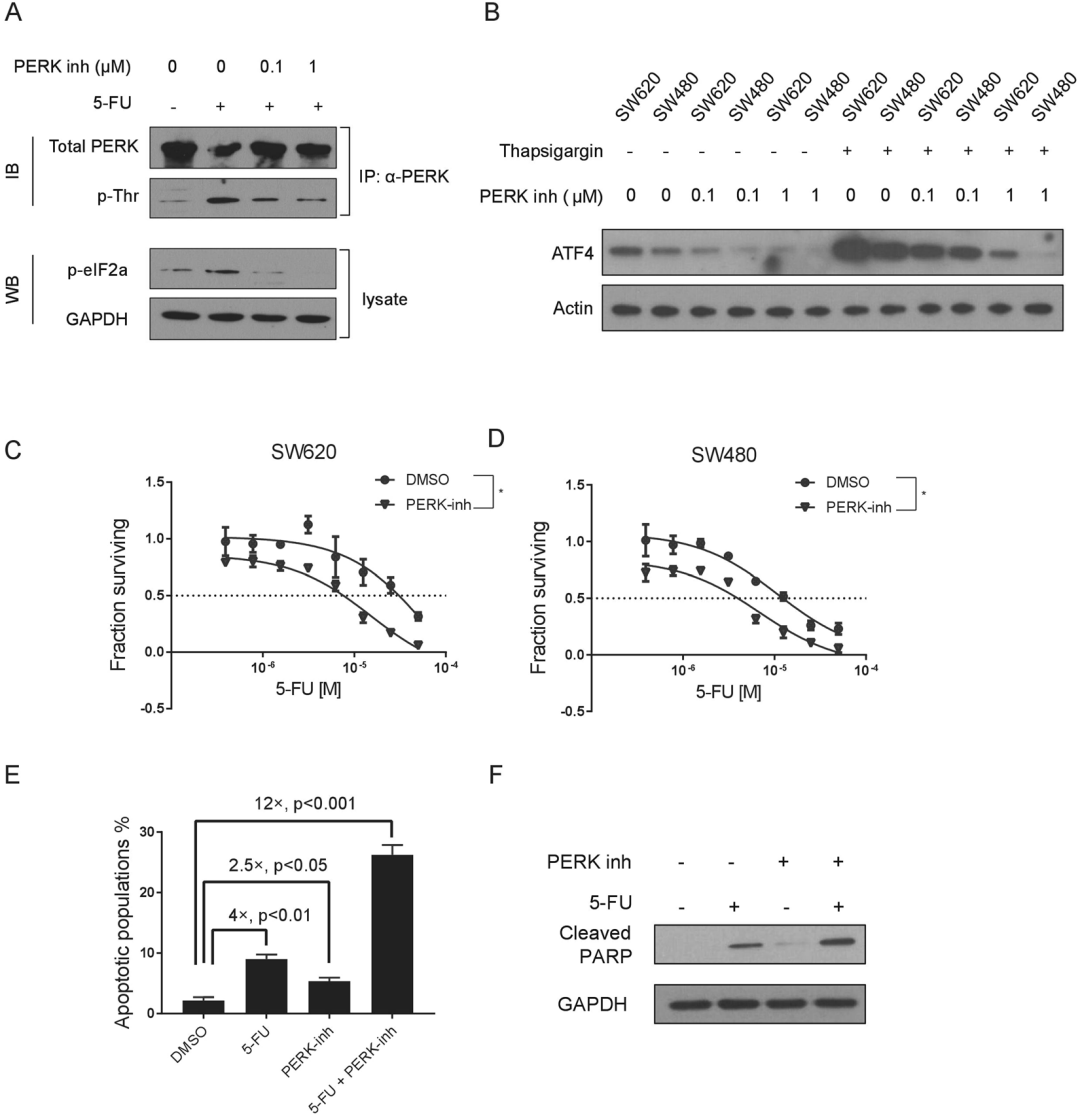
Chemical inhibition of PERK sensitizes colon cancer cells to 5-Fluorouracil treatment. (**A**) Total PERK protein was immunoprecipitated from cells as indicated, and western blotting was conducted to measure the level of phosphorylated PERK by use of a pan-pThr/Ser antibody. Cellular level of p-eIF2α was also measured for cells treated as indicated. (**B**) Western blotting showing the effects of an escalating dose of PERK inhibitor (0 µM, 0.1 µM and 1 µM) on ATF4 in SW620 and SW480 cells w/or w/o Thapsigargin treatment. β-Actin was used as loading control. (**C**,**D**) Dose response curves showing effects of the PERK inhibitor (1 µM) in sensitizing SW620 and SW480 cells to 5-FU treatment. (**E**) Flow cytometric analysis showing the percentage of apoptotic cells (SW620) treated as indicated. (**F)** Western blot showing the protein level of cleaved PARP which reflects the degree of cell apoptosis (SW620) treated as indicated. * means *P* < 0.05. Data are represented as mean ± SEM or the mean alone.

While shRNA-mediated gene expression reduction of the PERK pathway components was effective in sensitizing CRC cells to 5-FU, we next tested if chemical inhibition of the PERK pathway can produce the same effect. By use of a GSK-developed second generation small molecule inhibitor of PERK^20^, we can effectively suppress the activity of PERK in SW620 cells lines by measuring the level of phosphorylated PERK (Fig. 3A). The reduction of activity of the PERK pathway by this inhibitor can also be gauged by the level of its downstream factors (Figs 3B and S3A). Consistent with the findings from the shRNAs-based assays, treatment of the PERK inhibitor can effectively increase the sensitivity of CRC cells to 5-FU treatment (Figs 3C,D and S3B). To further confirm this finding, we measured apoptosis caused by 5-FU and PERK inhibitor in CRC cells. We found that treatment of PERK inhibitor significantly increases apoptotic cell death caused by 5-FU (Fig. 3E,F). As a control, inhibition of the IRE1 pathway does not sensitize CRC cells to 5-FU treatment (Fig. S3C). By use of a murine colon cancer cell line, CT26, we found that inhibition of the PERK activity also sensitizes CT26 cells to 5-FU treatment (Fig. S3D,E). Taking together, chemical inhibition of the PERK pathway sensitizes CRC cells to chemotherapy.

### Pharmacological inhibition of PERK potentiates treatment of 5-Fluorouracil *in vivo*

**Figure 4:**
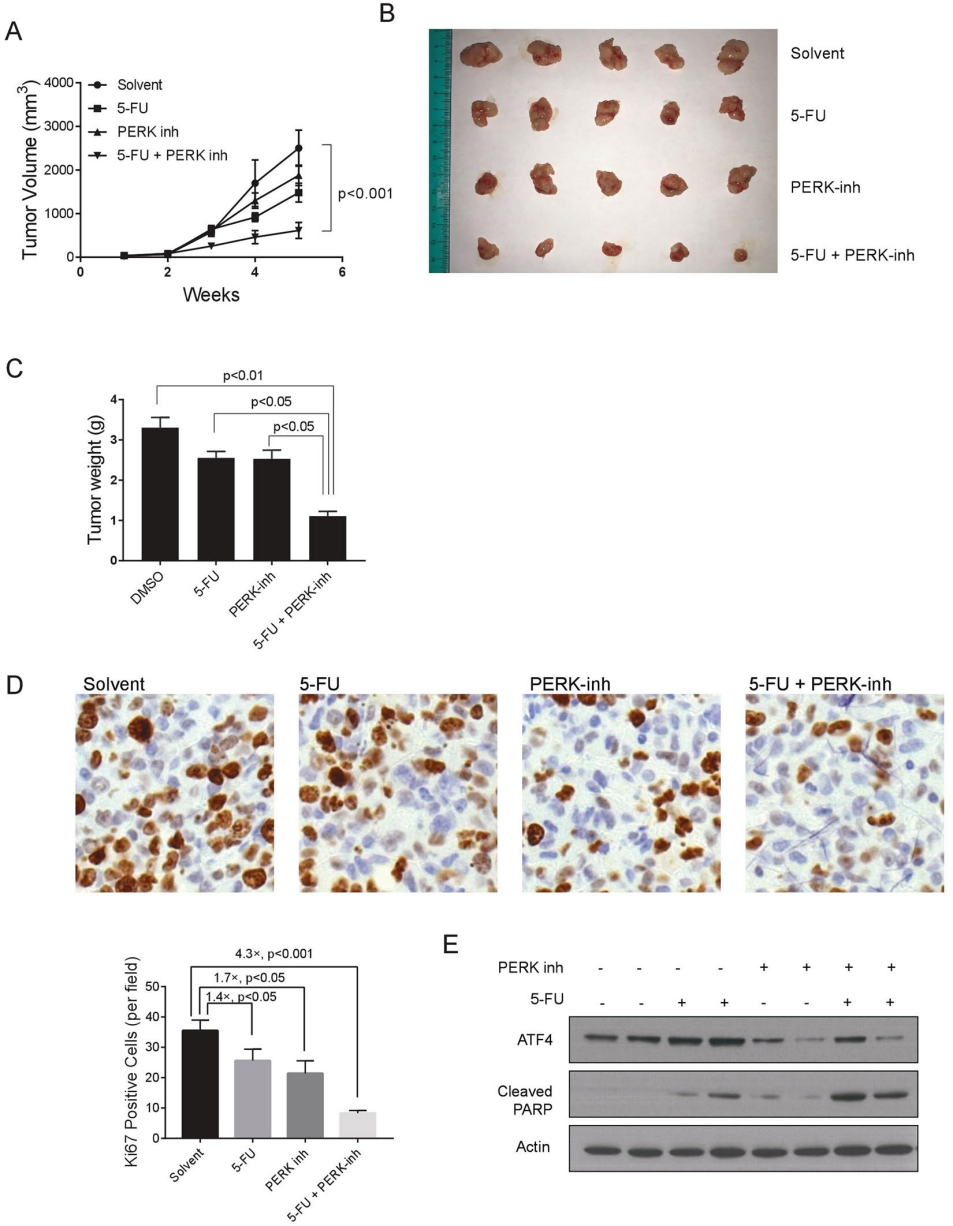
5-Fluorouracil and PERK inhibitor synergize to inhibit colon cancer *in vivo*. (**A**) (Tumor Volume) and (**B**) Tumors formed by SW620 cells in NOD/SCID mice treated with solvent control, 20 mg/kg 5-FU, 20 mg/kg PERK inhibitor, or combination of 5-FU and PERK inhibitor for 3 weeks. (**C**) Tumor weights of (**B**). (**D**) Ki67 staining for sectioning of tumors collected from (**B**). (**E**) Western blot showing the expression level of ATF4 and cleaved PARP of tumors collected from (**B**). β-Actin was used as loading control. Data are represented as mean ± SEM or the mean alone.

We next investigated if inhibition of PERK can synergize with chemotherapy *in vivo*. Consistent with prior studies, mono-treatment of 5-FU or PERK inhibitor, respectively, led to partial inhibition of tumor growth of the SW620 cells in nude mice (Fig. 4A–C). Remarkably, combo-treatment of 5-FU and PERK inhibitor exhibited a striking synergistic inhibition on tumor growth (Fig. 4A–C). We then examined the expression of Ki67, a classical marker of proliferation and cell growth, in the control, mono-treated and combo-treated tumors. We found the percentage of Ki67 positive cells was markedly decreased upon combined treatment with 5-FU and the PERK inhibitor (Fig. 4D). Lastly, we found that cleaved PARP was also increased in tumors treated with both PERK inhibitor and 5-FU (Fig. 4E), further showing the exceptional anti-tumor effects delivered by the combinatory administration with chemotherapeutic agent and PERK inhibitor.

## Discussion

Resistance to chemotherapy is a major hurdle in clinical management of late-stage colon cancer, as well as other types of human cancers. In this study, we set out to investigate the molecular mechanism of drug resistance of colon cancer cells, and discovered that activation of the PERK kinase-mediated stress signaling plays an important role in promoting cell survival under chemotherapy.

Activation of the unfolded protein response pathway has been implicated in cell survival. It has been reported that BiP, a classical chaperone protein induced by the UPR pathway, is required for breast cancer and lung cancer cells to grow and survive anti-cancer drugs^15^. This might be the underlying mechanism how two branches of the UPR signaling, the IRE1- and ATF6-led pathways, promote chemo-resistance of advanced cancers^21^. In addition, activation of the PERK pathway is required for breast cancer cells that have undergone an EMT to survive treatment of doxorubicin and paclitaxel, primarily through the NRF2 cascade, a non-canonical downstream of PERK^22^. Interestingly, while it was recently reported that the PERK-NRF2 pathway is activated and required for CRC cells to grow in the condition of ER stress and chemotherapy treatment^23^, we did not observe that NRF2 is differentially expressed in CRC cells with different sensitivity to 5-FU (data not shown). Instead, we found that expression of ATF4, a canonical downstream factor of the PERK kinase, is up-regulated in CRC cells that hold heightened resistance to chemotherapy drugs. We further showed that both PERK and ATF4 are functionally required for cell survival under 5-FU treatment. It is also possible that different downstream factors of PERK mediate resistance to different chemo-drugs.

The PERK-ATF4 signaling contributes to many cancer-related characteristics during disease progression. It has been reported that the PERK pathway is required for cell migration and invasion in breast and cervix cancers^24,25^. In addition, ATF4 promotes metastasis by reducing anoikis of cancer cells in suspension^26^. Consistent with its role in cancer cell migration and metastasis, the PERK branch of the UPR pathways is specifically activated in cancer cells that undergo an EMT, which is a key molecular program to drive metastasis^27^. Two recent studies suggested that the PERK pathway and its downstream factors promote metastasis through EMT-driven activation of the protein secretion pathways^28,29^. In addition to its role in migration and metastasis, the EMT molecular program is also closely related to drug resistance of cancer cells to chemotherapy and other anti-cancer regimens^30^. Therefore, our findings suggested that the PERK-ATF4 pathway may mediate the drug resistance caused by cell-state transition and plasticity program, e.g., the EMT program, in CRC. Since PERK is targetable by use of pharmacological inhibitors, it could be a promising approach to overcome drug resistance to CRC through inhibition of the PERK pathways. At the meantime, we noticed that other UPR-related mechanisms that underlie the drug resistance of CRC have been reported^31^. While CHOP, the downstream protein of ATF4 is expressed in prostate cancer cells even in the absence of ER stressors, CHOP is not expressed in the CRC cells that we tested. This tissue-specific CHOP expression might differentiate the role of PERK pathway in prostate cancer and colorectal cancer, because CHOP is largely known as a pro-apoptotic protein, while ATF4 is primarily pro-cancer development and progression. We also noticed that by use of the HCT116 cell model, another group has reported that the PKR kinase is responsible for the increase of p-eIF2α in 5-FU-treated colon cancer cells^32^. It suggests that colon cancer develop multiple stress-related mechanisms to resist 5-FU treatment, and different cell lines may apply different strategy in this process.

Cancer seems to acquire multiple malignant phenotypes at its late stage: recurrent and metastatic cancers are usually multidrug resistant, and enriched for cancer stem-like cells. One possibility would be that there are some central pathways that govern multiple cancer characteristics simultaneously. While we found that the PERK-ATF4 pathway is essential for drug-resistance of CRC cells, this pathway may also promote metastatic progression of CRC cells. It will be of great interest to examine if activation of the PERK-ATF4 pathway is also functionally required for migration and metastasis of CRC cells. Further investigation on this will open a window on an entirely new avenue of targeting cancer progression.

## Supplementary information

supplementary info file

## References

[1] Siegel RL, Miller KD, Jemal A (2017). Cancer Statistics, 2017. CA Cancer J Clin.

[2] Siegel RL (2017). Colorectal cancer statistics, 2017. CA Cancer J Clin.

[3] Sobrero AF (2008). EPIC: phase III trial of cetuximab plus irinotecan after fluoropyrimidine and oxaliplatin failure in patients with metastatic colorectal cancer. J Clin Oncol.

[4] Douillard JY (2013). Panitumumab-FOLFOX4 treatment and RAS mutations in colorectal cancer. N Engl J Med.

[5] Epple LM (2013). Induction of the unfolded protein response drives enhanced metabolism and chemoresistance in glioma cells. PLoS One.

[6] Lee E (2006). GRP78 as a novel predictor of responsiveness to chemotherapy in breast cancer. Cancer Res.

[7] Al-Rawashdeh FY, Scriven P, Cameron IC, Vergani PV, Wyld L (2010). Unfolded protein response activation contributes to chemoresistance in hepatocellular carcinoma. Eur J Gastroenterol Hepatol.

[8] Harding HP (2003). An integrated stress response regulates amino acid metabolism and resistance to oxidative stress. Mol Cell.

[9] Liu L, Wise DR, Diehl JA, Simon MC (2008). Hypoxic reactive oxygen species regulate the integrated stress response and cell survival. J Biol Chem.

[10] Chen JJ (2007). Regulation of protein synthesis by the heme-regulated eIF2alpha kinase: relevance to anemias. Blood.

[11] Ron D, Walter P (2007). Signal integration in the endoplasmic reticulum unfolded protein response. Nat Rev Mol Cell Biol.

[12] Hetz C (2012). The unfolded protein response: controlling cell fate decisions under ER stress and beyond. Nat Rev Mol Cell Biol.

[13] Kaufman RJ (2002). Orchestrating the unfolded protein response in health and disease. J Clin Invest.

[14] Walter P, Ron D (2011). The unfolded protein response: from stress pathway to homeostatic regulation. Science.

[15] Fu Y, Li J, Lee AS (2007). GRP78/BiP inhibits endoplasmic reticulum BIK and protects human breast cancer cells against estrogen starvation-induced apoptosis. Cancer Res.

[16] Grandi A (2016). ERMP1, a novel potential oncogene involved in UPR and oxidative stress defense, is highly expressed in human cancer. Oncotarget.

[17] Tang J (2012). CD147 induces UPR to inhibit apoptosis and chemosensitivity by increasing the transcription of Bip in hepatocellular carcinoma. Cell Death Differ.

[18] Stewart SA (2003). Lentivirus-delivered stable gene silencing by RNAi in primary cells. RNA.

[19] Wang Y (2000). Activation of ATF6 and an ATF6 DNA binding site by the endoplasmic reticulum stress response. J Biol Chem.

[20] Atkins C (2013). Characterization of a novel PERK kinase inhibitor with antitumor and antiangiogenic activity. Cancer Res.

[21] Gardner BM, Pincus D, Gotthardt K, Gallagher CM, Walter P (2013). Endoplasmic reticulum stress sensing in the unfolded protein response. Cold Spring Harb Perspect Biol.

[22] Del Vecchio CA (2014). De-differentiation confers multidrug resistance via noncanonical PERK-Nrf2 signaling. PLoS Biol.

[23] Salaroglio IC (2017). PERK induces resistance to cell death elicited by endoplasmic reticulum stress and chemotherapy. Mol Cancer.

[24] Nagelkerke A (2013). Hypoxia stimulates migration of breast cancer cells via the PERK/ATF4/LAMP3-arm of the unfolded protein response. Breast Cancer Res.

[25] Mujcic H (2013). Hypoxic activation of the PERK/eIF2alpha arm of the unfolded protein response promotes metastasis through induction of LAMP3. Clin Cancer Res.

[26] Dey S (2015). ATF4-dependent induction of heme oxygenase 1 prevents anoikis and promotes metastasis. J Clin Invest.

[27] Feng YX (2014). Epithelial-to-mesenchymal transition activates PERK-eIF2alpha and sensitizes cells to endoplasmic reticulum stress. Cancer Discov.

[28] Feng YX (2017). Cancer-specific PERK signaling drives invasion and metastasis through CREB3L1. Nat Commun.

[29] Howley, B. V., Link, L. A., Grelet, S., El-Sabban, M. & Howe, P. H. A CREB3-regulated ER-Golgi trafficking signature promotes metastatic progression in breast cancer. *Oncogene* (2017). 10.1038/s41388-017-0023-0 PMC5844805 29249802

[30] Gupta PB (2009). Identification of selective inhibitors of cancer stem cells by high-throughput screening. Cell.

[31] Rodvold, J. J. *et al*. Intercellular transmission of the unfolded protein response promotes survival and drug resistance in cancer cells. *Sci Signal* **10** (2017). 10.1126/scisignal.aah7177 PMC5962022 28588081

[32] Garcia MA (2011). The chemotherapeutic drug 5-fluorouracil promotes PKR-mediated apoptosis in a p53-independent manner in colon and breast cancer cells. PLoS One.

