# Supplementary material for: Activation of the PERK-ATF4 pathway promotes chemo-resistance in colon cancer cells
Source: Sci Rep. 2019 Mar 1;9:3210. doi: 10.1038/s41598-019-39547-x (PMC6397152; doi:10.1038/s41598-019-39547-x)

Title

Activation of the PERK-ATF4 pathway promotes chemo-resistance in colon cancer cells

Authors

Zhong Shi[#1], Xiaofu Yu[#2], Meiqin Yuan[1], Wangxia Lv[1], Tingting Feng[1], Rui Bai[3], Haijun Zhong[*1]

[#]The authors contributed equally to this work.

[1]Department of Medical Oncology, Zhejiang Cancer Hospital, Hangzhou 310022, China

[2]Department of Radiotherapy, Zhejiang Cancer Hospital, Hangzhou 310022, China

[3]Department of Medical Oncology, Hangzhou Cancer Hospital, Hangzhou 310000, China

Corresponding author:

Haijun Zhong, Tel +8657188122052, Fax +8657188122058, email address: zhonghj@zjcc.org.cn

Supplementary Figure Legends

Figure S1. Activation of the unfolded protein response pathways in colon cancer cells

(A) Realtime RT-PCR showing the relative expression level of ATF6 and GADD34 mRNA in six colon cancer cells. GAPDH was used as loading control. (B) Western blot showing the effects of Thapsigargin (100 nM) on PERK-ATF4 pathway and IRE1-sXBP1 in SW1116, Colo320DM and SW480 cells. GAPDH was used as loading control. (C) Luciferase reporter assay showing the effects of Thapsigargin (100 nM) on ATF6 activity in SW1116, Colo320DM and SW480 cells. Data are represented as mean ± SEM or the mean alone.

Figure S2. Correlation of the IRE1 and ATF6 pathway and sensitivities of colon cancer cells to 5-fluorouracil treatment

(A) and (B) and (C) Correlation analysis of the expression of spliced XBP-1, activity of ATF6 or the expression of BiP and EC50 of 5-FU in colon cancer cells. (D) Luciferase reporter assay showing the effects of 5-FU treatment on ATF6 activity in SW1116, Colo320DM and SW480 cells. (E) Western blot showing the effects of PERK knockdown on eIF2α expression in SW620 cells. Alpha Tubulin was used as loading control. (F) Western blot showing the effects of GCN2 and PKR knockdown on eIF2α and ATF4 expression in SW620 cells. GAPDH was used as loading control. (G) and (H) Western blot showing knockdown effects of shRNAs targeting ATF4 and IRE1 in SW620 cells. GAPDH was used as loading control. (I) Realtime RT-PCR showing knockdown effects of shRNAs targeting ATF6 in SW620 cells. GAPDH was used as loading control. (J) Dose response curves showing responses of SW620 cells transduced with a hairpin targeting Luciferase (shLuc) or two hairpins targeting IRE1 (shIRE1-1, shIRE1-2) to treatment of 5-FU. (K) Dose response curves showing responses of SW620 cells transduced with a hairpin targeting Luciferase (shLuc) or two hairpins targeting ATF6 (shATF6-1, shATF6-2) to treatment of 5-FU. Data are represented as mean ± SEM or the mean alone.

Figure S3. Chemical inhibition of PERK sensitizes colon cancer cells to 5-Fluorouracil treatment

(A) Western blot showing the effects of a PERK inhibitor (1 μM) on its downstream target eIF2α in SW620 and SW480 cells. GAPDH was used as loading control. (B) Dose response curves showing the effects of the PERK inhibitor (0.1 μM) in sensitizing SW620 cells to 5-FU treatment. (C) Dose response curves showing effects of the IRE1 inhibitor (10 μM) in sensitizing SW620 cells to 5-FU treatment. (D) CT26 cells were treated as indicated, and the level of total PERK, p-PERK, and p-eIF2α was measured by western blotting. (E) Dose response curves showing effects of the PERK inhibitor (0.1 μM) in sensitizing CT26 cells to 5-FU treatment. Data are represented as mean ± SEM or the mean alone.

Figure. S4 and S5

Raw data of the western blots used in this study.

Figure S1

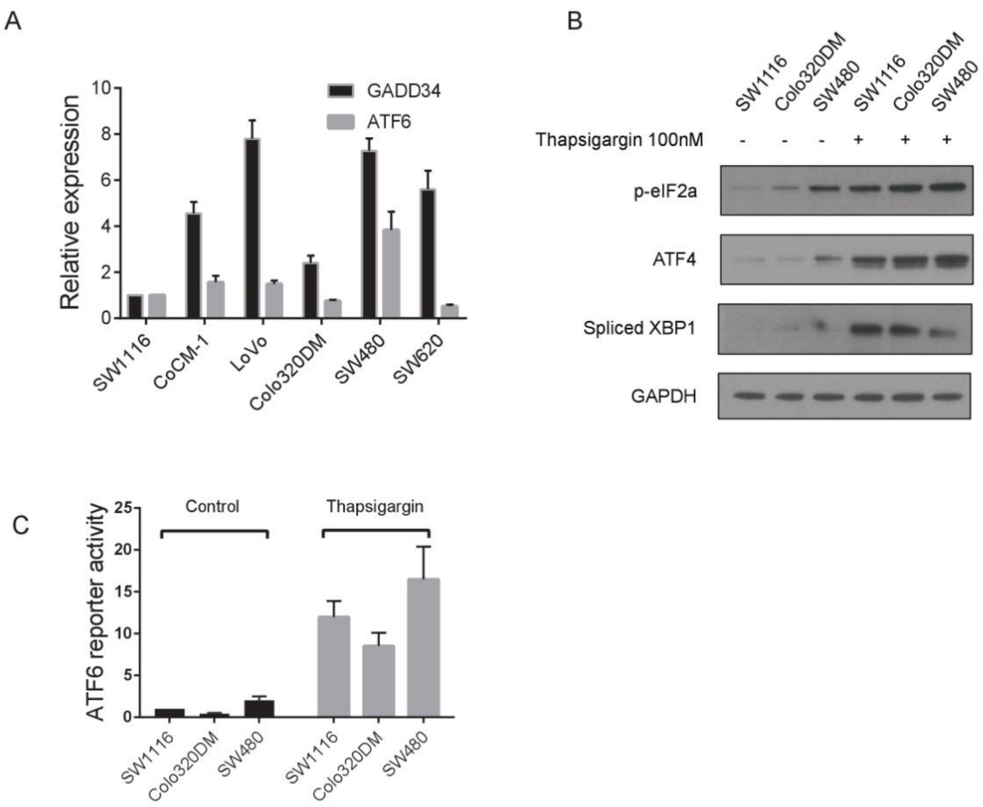
A
B
C
Relative expression
GADD34
ATF6
10
8
6
4
2
0
SW1116
CoCM-1
LoVo
Colo320DM
SW480
SW620
SW1116
Colo320DM
SW480
SW1116
Colo320DM
SW480
Thapsigargin 100nM
- - - + + +
p-eIF2a
ATF4
Spliced XBP1
GAPDH
ATF6 reporter activity
Control
Thapsigargin
25
20
15
10
5
0
SW1116
Colo320DM
SW480
SW1116
Colo320DM
SW480

Figure S2

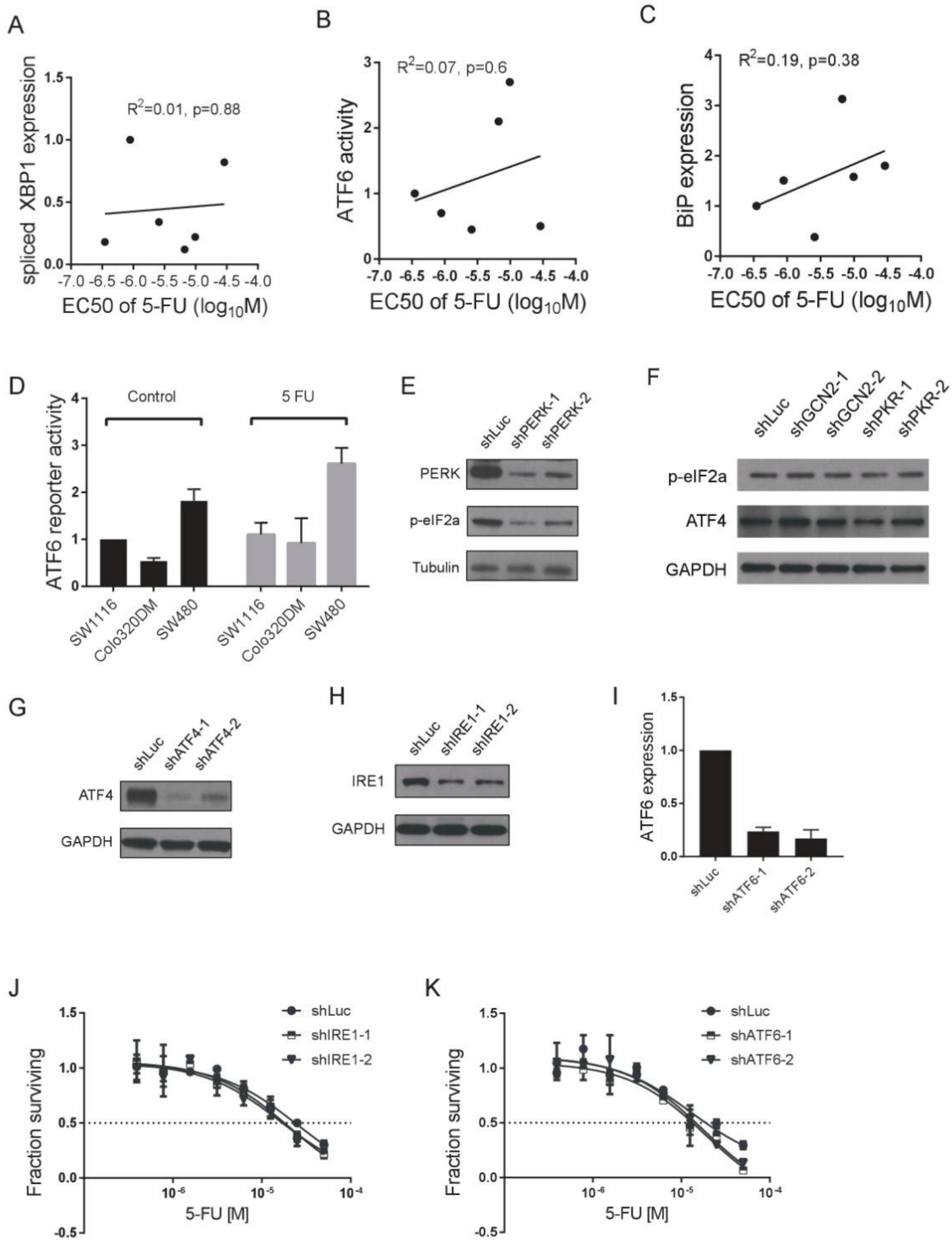
A
spliced XBP1 expression
R^2=0.01, p=0.88
EC50 of 5-FU (log10M)
B
ATF6 activity
R^2=0.07, p=0.6
EC50 of 5-FU (log10M)
C
BiP expression
R^2=0.19, p=0.38
EC50 of 5-FU (log10M)
D
ATF6 reporter activity
Control
5 FU
SW1116
Colo320DM
SW480
E
shLuc
shPERK-1
shPERK-2
PERK
p-eIF2a
Tubulin
F
shLuc
shGCN2-1
shGCN2-2
shPKR-1
shPKR-2
p-eIF2a
ATF4
GAPDH
G
shLuc
shATF4-1
shATF4-2
ATF4
GAPDH
H
shLuc
shIRE1-1
shIRE1-2
IRE1
GAPDH
I
ATF6 expression
shLuc
shATF6-1
shATF6-2
J
Fraction surviving
5-FU [M]
shLuc
shIRE1-1
shIRE1-2
K
Fraction surviving
5-FU [M]
shLuc
shATF6-1
shATF6-2

Figure S3

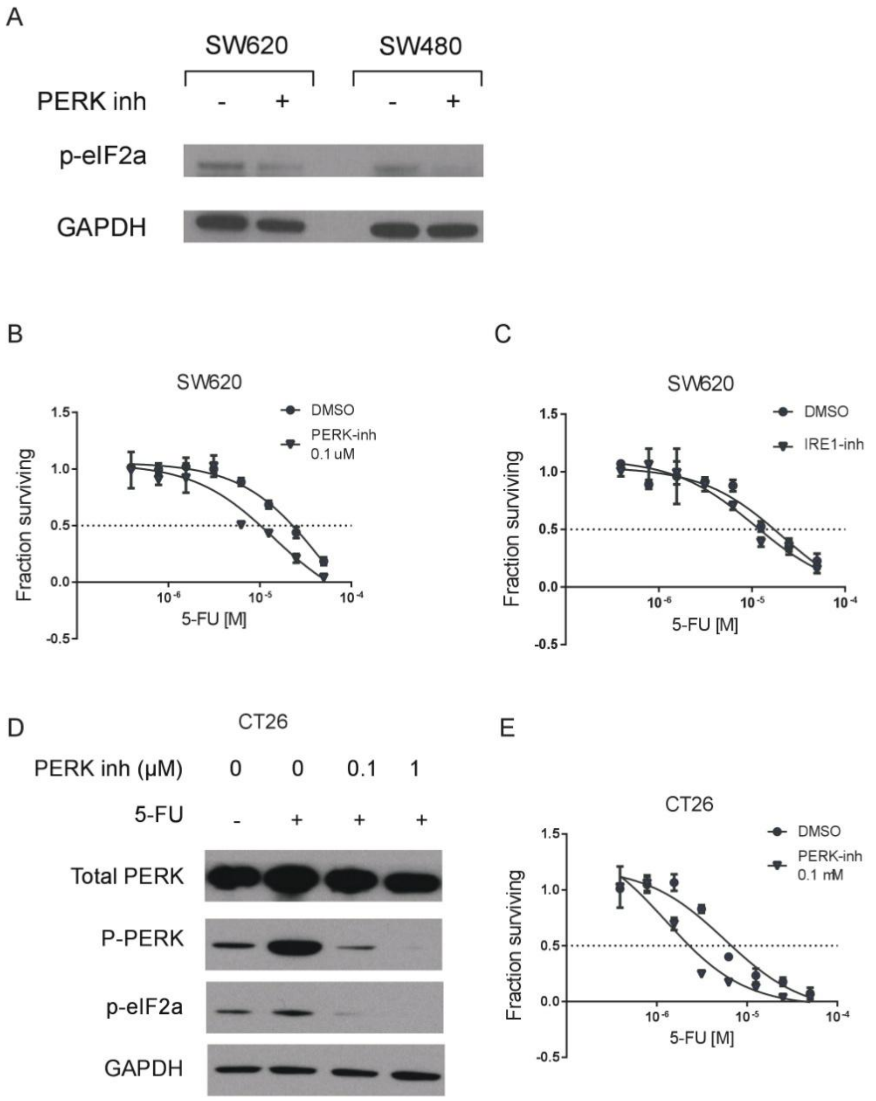
A
SW620
SW480
PERK inh
-
+
-
+
p-eIF2a
GAPDH
B
SW620
DMSO
PERK-inh
0.1 uM
Fraction surviving
5-FU [M]
C
SW620
DMSO
IRE1-inh
Fraction surviving
5-FU [M]
D
CT26
PERK inh (µM)
0
0
0.1
1
5-FU
-
+
+
+
Total PERK
P-PERK
p-eIF2a
GAPDH
E
CT26
DMSO
PERK-inh
0.1 mM
Fraction surviving
5-FU [M]

Figure S4

This blot is used in Figure 1A (PERK).

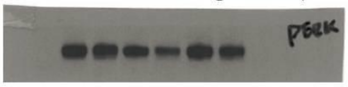

This blot is used in Figure 1A (p-eIF2α).

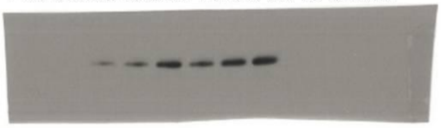

This blot is used in Figure 1A (total p-eIF2α).

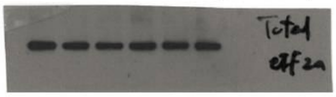

This blot is used in Figure 1A (ATF4).

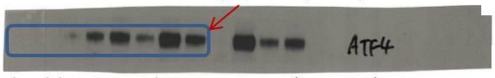

This blot is used in Figure 1A (GAPDH).

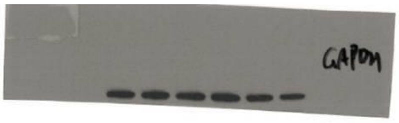

This blot is used in Figure 2E (p-eIF2α).

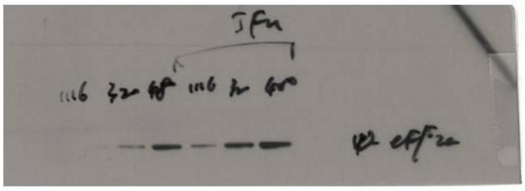

This blot is used in Figure 1B (IRE1).

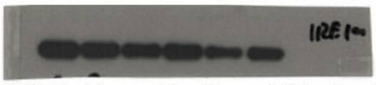

This blot is used in Figure 1B (spliced XBP1).

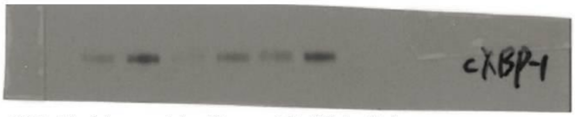

This blot is used in Figure 1B (Tubulin).

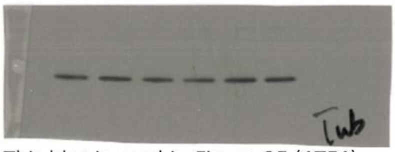

This blot is used in Figure 2E (ATF4).

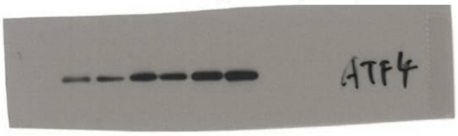

This blot is used in Figure 2E (spliced XBP1).

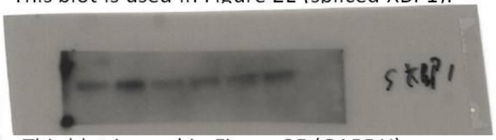

This blot is used in Figure 2E (GAPDH).

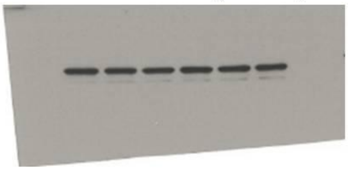

Figure S5

This blot is used in Figure 3A (ATF4).

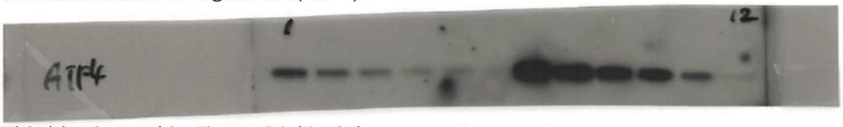

This blot is used in Figure 3A (Actin).

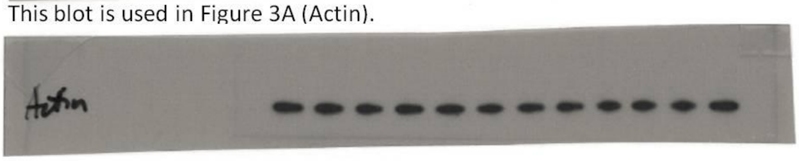

This blot is used in Figure 3E (cleaved PARP).

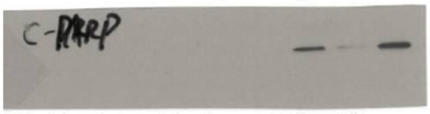

This blot is used in Figure 3E (GAPDH).

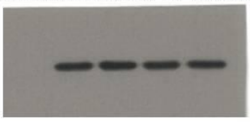

This blot is used in Figure 4E (ATF4).

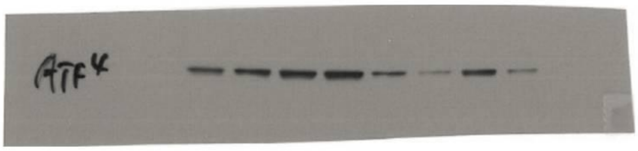

This blot is used in Figure 4E (cleaved PARP).

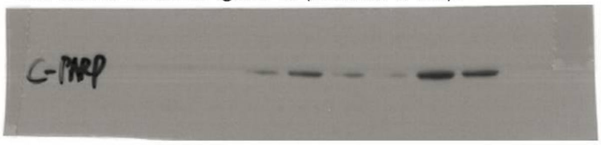

This blot is used in Figure 4E (Actin).

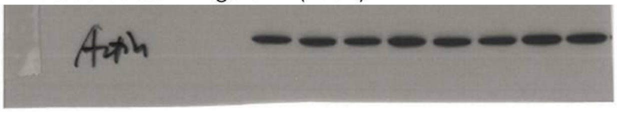

Supplement: Supplementary file 1 — supplementary info file [file 41598_2019_39547_MOESM1_ESM.pdf]
